# Healthcare costs associated with cardiovascular events in patients with hyperlipidemia or prior cardiovascular events: estimates from Swedish population-based register data

**DOI:** 10.1007/s10198-015-0702-0

**Published:** 2015-06-16

**Authors:** S. Hallberg, S. R. Gandra, K. M. Fox, J. Mesterton, J. Banefelt, G. Johansson, L.-Å. Levin, P. Sobocki

**Affiliations:** Quantify Research, Hantverkargatan 8, 112 21 Stockholm, Sweden; Amgen Inc., Thousand Oaks, CA USA; Strategic Healthcare Solutions, LLC, Baltimore, MD USA; LIME/Medical Management Centre, Karolinska Institute, Stockholm, Sweden; Department of Public Health and Caring Sciences, Uppsala University, Uppsala, Sweden; Department of Medical and Health Sciences, Linköping University, Linköping, Sweden; IMS Health, Stockholm, Sweden

**Keywords:** Cardiovascular, Costs, Hyperlipidemia, CVD, Resource use, Burden of illness, I12

## Abstract

**Objectives:**

To estimate healthcare costs of new cardiovascular (CV) events (myocardial infarction, unstable angina, revascularization, ischemic stroke, transient ischemic attack, heart failure) in patients with hyperlipidemia or prior CV events.

**Methods:**

A retrospective population-based cohort study was conducted using Swedish national registers and electronic medical records. Patients with hyperlipidemia or prior CV events were stratified into three cohorts based on CV risk level: history of major cardiovascular disease (CVD), coronary heart disease (CHD) risk-equivalent, and low/unknown risk. Propensity score matching was applied to compare patients with new events to patients without new events for estimation of incremental costs of any event and by event type.

**Results:**

A CV event resulted in increased costs over 3 years of follow-up, with the majority of costs occurring in the 1st year following the event. The mean incremental cost of patients with a history of major CVD (*n* = 6881) was €8588 during the 1st year following the event. This was similar to that of CHD risk-equivalent patients (*n* = 3226; €6663) and patients at low/unknown risk (*n* = 2497; €8346). Ischemic stroke resulted in the highest 1st-year cost for patients with a history of major CVD and CHD risk-equivalent patients (€10,194 and €9823, respectively); transient ischemic attack in the lowest (€3917 and €4140). Incremental costs remained elevated in all cohorts during all three follow-up years, with costs being highest in the major CVD history cohort.

**Conclusions:**

Healthcare costs of CV events are substantial and vary considerably by event type. Incremental costs remain elevated for several years after an event.

**Electronic supplementary material:**

The online version of this article (doi:10.1007/s10198-015-0702-0) contains supplementary material, which is available to authorized users.

## Introduction

Cardiovascular disease (CVD), with the usual underlying pathology of atherosclerosis, is a major cause of premature death worldwide and a substantial source of disability. Consequently, CVD contributes extensively to the escalating costs of healthcare [1]. The most common manifestation of CVD is coronary heart disease (CHD). CHD has been estimated to be the leading cause of disability in Europe, accounting for approximately 10 % of total disability-adjusted life years [2].

There are several recent studies that have examined the costs of CVD-related events, and especially so with a focus on short-term healthcare costs due to CVD-related events [3–10]. These studies have not included patients based on a diagnosis of or treatment for hyperlipidemia but instead included patients hospitalized for CV events [3, 10, 11], patients with atherosclerosis [4], hypertension [5], or acute coronary syndrome [6, 7, 12], or used a prevalence-based approach [8, 9]. Only a few studies have examined the long-term costs associated with CVD-related events [3, 10, 11] and stratified by specific event types [13]. There are limited data that have focused on costs of CVD-related events over the acute phase, short term and long term for hyperlipidemia patients specifically. Also, estimates of the cost of recurrent and subsequent CV events are limited; previous studies have focused on the first CV event and limited the study cohorts to those without CVD at baseline.

The present study contributes to filling the gap in available evidence in the area by providing acute, short-term and long-term direct incremental costs in hyperlipidemia patients. In addition, costs stratified by CV event type and costs of subsequent CV events are provided. These costs may serve as an important basis for health economic analysis to estimate the cost-effectiveness of health technologies aimed at preventing CVD-related events.

The objective of the study was to characterize the use of healthcare resources and to estimate the acute (first 30 days), the short-term (1st year), and long-term (up to 3 years) healthcare costs of new CV events (myocardial infarction, unstable angina pectoris, revascularization [percutaneous transluminal coronary angioplasty], ischemic stroke, transient ischemic attack, or heart failure) in patients with hyperlipidemia or a history of CV events.

## Methods

### Study design and population

This was a retrospective register study based on a matched control design. The primary data sources for the study were electronic medical records in primary care and three selected national compulsory health registers which are governed by the National Board of Health and Welfare. By merging data from the medical records with data from the National Patient Register, the Cause of Death Register, and the Swedish Prescribed Drug Register, information on a patient-related basis was available for the following resource and cost domains:

- Pharmaceuticals: the Prescribed Drug Register collects data on the total costs associated with each filled prescription.
- Inpatient and outpatient care: all hospitalizations, surgical procedures, and outpatient specialist visits were collected from the National Patient Register for the complete observable period for each patient.
- Primary care: all physical contacts and contacts by phone with nurses, general practitioners, and other healthcare personnel in the primary care centers were collected.
- Death: the Cause of Death Register provided confirmed dates of death, allowing for censoring of patients.

Unique individual patient ID numbers were available in all data sources which allowed for linkage of individual patients between data sets. The linkage of de-identified individual patients was performed by the National Board of Health and Welfare. The study was approved by the Swedish Ethics Review Board.

Patients were included in the study population based on having hyperlipidemia, defined as treatment with lipid-lowering therapy (LLT) as this is the most accurate way of identifying hyperlipidemia patients in Sweden. Patients were included on the date of the first prescription of LLT during 2006 if a second filled prescription followed within 6 months. The study inclusion date was defined as the date of the first of the filled prescriptions for LLT. Additionally, patients without LLT during 2006 but with a prior history of CV events (within the past 5 years) were also included in the study in order to capture patients with CVD at baseline. For patients that did not receive LLT but who had a history of CV events, the study inclusion date was defined as January 1, 2006.

Patients were stratified into three separate cohorts based on CVD risk according to the National Cholesterol Education Program Adult Treatment Panel III guidelines [14] and the European Society of Cardiology guidelines [15] from 10 years prior to and up until study inclusion. The purpose of the stratification was to analyze health resource utilization (HRU) and costs over different sub-groups of patients with varying risk of CVD. The stratification was done according to the following definitions:

1. History of major CVD cohort: prior diagnosis of myocardial infarction, unstable angina pectoris, ischemic stroke, or revascularization.
2. CHD risk equivalent cohort: patients not included in the history of major CVD cohort and with prior diagnosis of diabetes, peripheral artery disease, abdominal aortic aneurysm, transient ischemic attack, or stable angina pectoris.
3. Low/unknown risk cohort: patients not included in the history of major CVD cohort or CHD risk equivalent cohort.

Patients were observed from study inclusion until December 31, 2009 for identification of new CV events (exposure time). The first new CV event during this period was defined as the index date. Patients were followed for 3 years (up until December 31, 2012) after the index date (or until death, whichever occurred first) for identification of HRU related to CV events on an individual patient level.

Direct costs were estimated based on HRU related to a new CV event which consisted of the number of healthcare visits, hospitalization days, surgical procedures, and pharmaceutical use on an individual patient level. Hospitalizations, procedures or healthcare visits were required to have an accompanying primary diagnosis or any surgery code of a CV event and pharmaceuticals were required to be LLT in order to be defined as related to CV events. Costs were based on a review of Swedish regional price lists from 2012 from different healthcare regions in Sweden [16–21]. Cost outcomes were calculated for the following time periods:

1. 365–1 days before new CV event (pre-index period),
2. 0–30 days after new CV event (acute period),
3. 0–365 days after new CV event (short term),
4. 366–730 days after new CV event, and
5. 731–1095 days after new CV event (long term).

### Statistical analysis

In order to estimate HRU and costs of new CV events, a comparison between patients with and without a new CV event within each CVD risk level was conducted. To control for confounders and limit bias, propensity score matching was applied. Patients without a CV event between study inclusion and December 31, 2009 within each CVD risk level were matched to patients with a CV event within the same CVD risk category based on a 1:1 match without replacement. The covariates included in the matching were age, gender, Charlson comorbidity index, hospital visits related to CV events during the 1-year period prior to study inclusion, and days of hospitalization related to CV events during the 1-year period prior to study inclusion. The propensity score was estimated using logistic regression and the caliper approach was used for matching. The caliper method uses a tolerance level of the maximum propensity score distance to avoid the risk of bad matches. The maximum propensity score distance was set to a fourth of the standard deviation of the propensity score. The index date for patients without a new CV event was set to the index date of the patient to whom they were matched. The quality of the matching was evaluated by investigating the standardized differences between cases and matched controls in the variables used in the matching. An ex-ante limit of 10 % in standardized differences was set for all variables used in the matching.

Costs were estimated by multiplying each resource use with the corresponding average unit cost from the price lists. All costs were converted to 2012 euro (€) from 2012 Swedish kronor (conversion rate: 1 euro = 8.71 Swedish kronor [22]). Incremental costs associated with a new CV event were calculated as the mean difference in costs between the matched patients with and without new CV events within each cohort.

The non-parametric Wilcoxon signed-ranked test was used to test for statistical significance in differences in costs between patients with new events and matched patients without events, for each time period and each cohort.

All data management and statistical analysis was performed using MySQL and Stata 12.

## Results

### Patient attrition and characteristics

A total of 96,256 patients were identified for inclusion in the study. Of these, 14,008 (14.6 %) experienced a CV event during exposure time. Outcomes were assessed for the 12,604 (90.0 %) patients for whom a match could be found based on characteristics at study inclusion. Cases (i.e., patients with a new event during exposure time) and matched controls (i.e., patients without a new event during exposure time) were followed for calculation of HRU and costs in parallel from the index date.

**Fig. 1:**
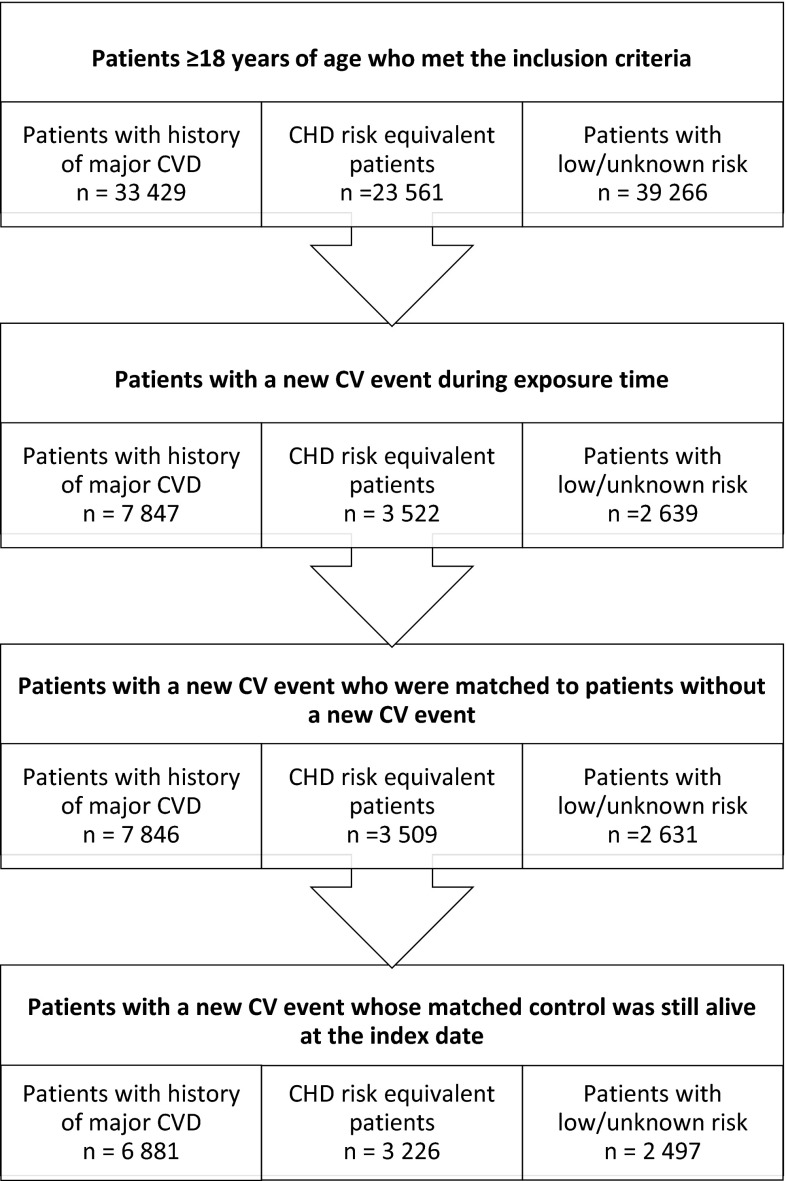
Patient attrition

Among patients with new CV events, 6881 of the included patients had a history of major CVD, 3226 patients belonged to the CHD risk equivalent cohort and the remaining 2497 patients to the low/unknown risk cohort (Fig. 1). Equally many patients without new CV events were included as controls, making the total cohort sizes twice as large (13,796 patients in the history of major CVD, 6448 patients in the CHD risk equivalent cohort and 4994 patients in the low/unknown risk cohort). The characteristics of the different cohorts at index date are presented in Table 1, stratified by patients with and without new CV events.

**Table 1:** Patient baseline characteristics at index date for cases (matched patients with new cardiovascular events) and controls (matched patients without new cardiovascular events)

Patient baseline characteristics	Cases	Controls
History of major CVD cohort (*n* = 6881)		
Mean age (SD)	75.26 (11.03)	75.36 (10.86)
Gender (% females)	39.2	38.8
Mean days of follow-up (SD)	741 (430)	741 (430)
Mean Charlson comorbidity index (SD)*	2.81 (2.11)	2.62 (2.12)
Myocardial infarction (%)*	43	38
Peripheral vascular disease (%)*	15	11
Cerebrovascular disease (%)*	42	46
Diabetes mellitus (%)*	25	21
Lipid-lowering therapy (%)	63.2	62.3
Statin treatment (%)	60.0	59.7
CHD risk equivalent cohort (*n* = 3226)		
Mean age (SD)**	74.47 (10.29)	74.61 (10.38)
Gender (% females)	43.6	43.1
Days of follow-up (SD)	800 (413)	800 (413)
Mean Charlson comorbidity index (SD)*	2.59 (2.12)	2.39 (2.12)
Myocardial Infarction (%)*	1	0
Peripheral vascular disease (%)*	18	14
Cerebrovascular disease (%)*	20	17
Diabetes mellitus (%)	41	41
Lipid-lowering therapy (%)*	78.8	83.1
Statin treatment (%)*	74.6	78.8
Low/unknown risk cohort (*n* = 2497)		
Mean age (SD)***	73.09 (11.00)	73.30 (10.96)
Gender (% females)	46.3	46.0
Days of follow-up (SD)	856 (391)	856 (391)
Mean Charlson comorbidity index (SD)*	1.20 (1.55)	1.04 (1.56)
Myocardial infarction (%)**	1	0
Peripheral vascular disease (%)**	4	3
Cerebrovascular disease (%)*	9	6
Diabetes mellitus (%)*	4	2
Lipid-lowering therapy (%)*	73.8	80.2
Statin treatment (%)*	69.9	75.8

* *p* value <0.01, ** *p* value <0.05, *** *p* value <0.10

Many of the patterns between cases and controls were seen in all three cohorts. Cases and controls in all risk cohorts were relatively balanced in terms of demographic and background characteristics, although cases were slightly more comorbid than the controls at index. There did not appear to be any systematic differences between cases and controls in LLT around the time of index for patients with a history of major CVD. For the other cohorts, a higher share of controls was on LLT compared to cases in the same cohort.

Patients with a history of major CVD were older and more comorbid compared to the other cohorts. Despite this, the history of major CVD cohort had the lowest share of patients on LLT amongst the three risk cohorts.

### Health resource utilization and associated costs

Days of CV-related hospitalization, number of CV-related outpatient visits, and number of primary care contacts are presented as separate categories of HRU (Table 2).

**Table 2:** Mean health resource utilization for cases (matched patients with new cardiovascular events) and controls (matched patients without new cardiovascular events)

	History of major CVD cohort			CHD risk equivalent cohort			Low/unknown risk cohort		
	Mean HRU (SD)			Mean HRU (SD)			Mean HRU (SD)		
	*n*	Cases	Controls	*n*	Cases	Controls	*n*	Cases	Controls
Hospitalization (days)									
365–1 days before new CV event	6881	1.37 (5.76)	0.91 (4.94)	3226	0.32(2.29)	0.16 (1.69)	2497	0.34 (2.62)	0.13 (1.69)
0–365 days after new CV event	6881	9.70 (11.20)	0.17 (2.43)	3226	9.64 (11.91)	0.07 (1.20)	2497	8.85 (11.15)	0.03 (0.54)
366–730 days after new CV event	6251	1.92 (7.91)	0.14 (1.60)	2995	1.84 (7.57)	0.08 (1.18)	2331	1.15 (5.01)	0.10 (1.49)
731–1095 days after new CV event	4987	1.62 (6.33)	0.32 (2.84)	2465	1.36 (5.50)	0.34 (3.64)	2031	1.06 (5.10)	0.18 (1.76)
Outpatient (visits)									
365–1 days before new CV event	6881	0.12 (0.54)	0.06 (0.38)	3226	0.09 (0.39)	0.04 (0.55)	2497	0.10 (0.45)	0.03 (0.25)
0–365 days after new CV event	6881	0.22 (0.78)	0.03 (0.23)	3226	0.28 (1.15)	0.02 (0.16)	2497	0.27 (0.68)	0.01 (0.16)
366–730 days after new CV event	6251	0.13 (0.54)	0.03 (0.22)	2995	0.14 (0.57)	0.02 (0.15)	2331	0.18 (1.67)	0.02 (0.16)
731–1095 days after new CV event	4987	0.09 (0.42)	0.03 (0.36)	2465	0.11 (0.45)	0.02 (0.17)	2031	0.10 (0.44)	0.02 (0.17)
Primary care (contacts)									
365–1 days before new CV event	6881	2.19 (4.19)	1.88 (3.61)	3226	2.65 (4.64)	2.21 (3.97)	2497	1.85 (3.95)	1.47 (3.09)
0–365 days after new CV event	6881	1.93 (4.15)	1.50 (3.37)	3226	2.44 (4.57)	1.86 (3.42)	2497	1.87 (3.97)	1.30 (2.91)
366–730 days after new CV event	6251	1.91 (4.03)	1.66 (3.44)	2995	2.30 (4.44)	2.15 (3.77)	2331	1.79 (4.00)	1.46 (3.22)
731–1095 days after new CV event	4987	1.78 (3.97)	1.57 (3.41)	2465	2.20 (4.68)	2.01 (3.63)	2031	1.66 (3.86)	1.34 (2.98)

Cases and controls were somewhat unbalanced at index date, as HRU during the pre-index period (365–1 days before the new CV event) was higher among cases than controls for all categories, despite having been matched on outpatient specialist visits and days of hospitalization during the 1-year period prior to study inclusion. There was a sharp increase among cases in all categories of HRU during the 1-year period after index for the history of major CVD cohort, with the exception of primary care contacts. Notably, the mean number of hospitalization days in the history of major CVD cohort increased from 1.37 during the 1-year period before index date to 9.70 during the year after index. This increase was also seen in the CHD risk equivalent (from 0.32 to 9.64 days) and low/unknown risk cohorts (from 0.34 to 8.84 days). There was no such corresponding increase seen among controls in any of the three cohorts, as HRU of controls was, in general, stable across all periods.

Total costs associated with new CV events are presented in 2012 euro and by risk cohort for all time periods (Table 3).

**Table 3:** Cost of health resource utilization by cardiovascular risk cohort for cases (matched patients with new cardiovascular events) and controls (matched patients without new cardiovascular events)

	Mean costs of HRU related to CV events in €, for all cohorts (SD)			
	*n*	Cases	Controls	Incremental*
History of major CVD cohort				
365–1 days before new CV event	6881	2219 (4722)	1630 (4094)	589 (5623)
0–30 days after new CV event	6881	6459 (5471)	94 (469)	6365 (5488)
0–365 days after new CV event	6881	9489 (8398)	902 (2269)	8588 (8624)
366–730 days after new CV event	4987	2672 (6367)	940 (1836)	1732 (6602)
731–1095 days after new CV event	4154	2384 (5157)	1056 (2554)	1328 (5772)
CHD risk equivalent cohort				
365–1 days before new CV event	3226	1489 (2314)	1341 (7119)	148 (7292)
0–30 days after new CV event	3226	6397 (5382)	116 (875)	6281 (5450)
0–365 days after new CV event	3226	9811 (9167)	1148 (6879)	8663 (11,482)
366–730 days after new CV event	2465	2835 (6072)	1312 (5795)	1523 (8365)
731–1095 days after new CV event	2160	2474 (4841)	1460 (6305)	1014 (8005)
Low/unknown risk cohort				
365–1 days before new CV event	2497	1029 (2152)	699 (1473)	330 (1897)
0–30 days after new CV event	2497	6524 (5616)	54 (130)	6470 (5613)
0–365 days after new CV event	2497	8925 (8192)	579 (857)	8346 (8234)
366–730 days after new CV event	2031	1905 (4122)	677 (1359)	1228 (4320)
731–1095 days after new CV event	1841	1771 (4256)	725 (1819)	1046 (4663)

* *p* value <0.01 for all incremental costs

The mean costs of HRU were higher for cases than for matched controls across all time periods and cohorts, including in the pre-index period. This difference was small in the CHD risk equivalent cohort, however (a difference of €148 in the pre-index period compared to €589 and €330 in the history of major CVD cohort and the low/unknown risk cohort, respectively). A sharp increase in mean costs of cases in all cohorts could be seen right after the index event with acute costs in the range of €6397–€6524, after which costs decreased during the subsequent years. No corresponding increase was observed in controls, with mean costs of controls remaining relatively stable during the entire 3-year period. The short-term costs of patients with new events were similar across the three cohorts (costs in the range of €8925–€9811).

The long-term costs were also similar in the history of major CVD and CHD risk equivalent cohorts (€2384 and €2474, respectively) but lower for the low/unknown risk cohort (€1771). Costs remained elevated 2–3 years after index compared to the pre-index period in all three cohorts. The history of major CVD cohort saw the greatest regression toward levels close to those seen before index (the difference between pre-index and long-term costs was €165 for cases in the history of major CVD cohort; corresponding figures for the CHD risk equivalent and low/unknown risk cohort were €985 and €742, respectively).

The incremental costs between matched patients with and without new CV events were found to be statistically significant in all time periods for all cohorts (Table 3). The incremental mean cost during the 1st year following a new CV event was €8588 for patients with a history of major CVD, similar to the cost of patients in the CHD risk equivalent cohort (€8663) and higher than the cost of the low/unknown risk cohort (€7901). Patients in the history of major CVD cohort had the highest incremental costs during both the 2nd and 3rd year after a new CV event (€1733 and €1328, respectively). The CHD risk equivalent cohort had higher incremental costs than the low/unknown risk cohort during the 2nd year (€1523 vs €1162), whilst the incremental costs during the 3rd year following a new CV event became more alike (€1014 for the CHD risk equivalent and €990 for the low/unknown risk cohort).

Total costs of HRU following a new event were also analyzed by type of new event (Table 4).

**Table 4:** Cost of health resource utilization by cohort and event type for cases (matched patients with new cardiovascular events) and controls (matched patients without new cardiovascular events)

	CVD history cohort: mean costs of HRU in € (SD)				CHD risk equivalent cohort: mean costs of HRU in € (SD)			
	*n*	Cases	Controls	Incremental	*n*	Cases	Controls	Incremental
Event type: heart failure								
365–1 days before new CV event	1763	2762 (5452)	2033 (5314)	729* (6929)	924	2090 (3430)	1950 (13,124)	140* (13,261)
0–365 days after new CV event	1763	9137 (9452)	920 (2333)	8217* (9649)	924	9730 (10,035)	1470 (12,667)	8259* (16,278)
366–730 days after new CV event	1053	3871 (7321)	955 (2053)	2917* (7521)	597	4498 (9399)	1717 (11,416)	2781* (14,805)
366–1095 days after new CV event	1053	6299 (9751)	1682 (3411)	4617* (10,321)	597	6988 (11,240)	3257 (22,093)	3731* (24,976)
731–1095 days after new CV event	764	3346 (6463)	1002 (2262)	2344* (6956)	465	3197 (5404)	1977 (12,316)	1220* (13,612)
Event type: ischemic stroke								
365–1 days before new CV event	1613	2020 (5371)	1475 (3504)	545* (5672)	667	1263 (1773)	1101 (1306)	162*** (2173)
0–365 days after new CV event	1613	11,040 (9049)	847 (1895)	10,194* (9223)	667	10,837 (10,460)	1014 (1578)	9823* (10,564)
366–730 days after new CV event	1146	2265 (6191)	983 (1940)	1282* (6510)	504	2237 (4493)	1172 (1365)	1064* (4702)
366–1095 days after new CV event	1146	4173 (8937)	1879 (3532)	2294* (9446)	504	4480 (7694)	2364 (3166)	2116* (8475)
731–1095 days after new CV event	940	2326 (6403)	1093 (2496)	1233* (6791)	458	2468 (5558)	1311 (2655)	1157* (6267)
Event type: myocardial infarction								
365–1 days before new CV event	1868	2010 (4016)	1536 (3641)	474* (4846)	798	1286 (1622)	1159 (1399)	126*** (2104)
0–365 days after new CV event	1868	9672 (8177)	871 (2709)	8801* (8446)	798	10,820 (9202)	1013 (1458)	9807* (9195)
366–730 days after new CV event	1303	2540 (5081)	961 (2151)	1579* (5545)	594	2784 (5394)	1162 (1497)	1622* (5569)
366–1095 days after new CV event	1303	4525 (7040)	1910 (4264)	2615* (8353)	594	4917 (7244)	2248 (3347)	2669* (7870)
731–1095 days after new CV event	1087	2380 (4254)	1138 (3411)	1242* (5521)	511	2479 (4465)	1262 (2766)	1217* (5205)
Event type: transient ischemic attack								
365–1 days before new CV event	469	1597 (4106)	1340 (3962)	257 (5564)	266	1181 (1639)	1090 (1432)	91 (2057)
0–365 days after new CV event	469	4888 (5549)	971 (1516)	3917* (5783)	266	5172 (6374)	1032 (1292)	4140* (6480)
366–730 days after new CV event	405	2544 (10,287)	921 (1189)	1623* (10,260)	236	1981 (3684)	1250 (2397)	731* (4455)
366–1095 days after new CV event	405	3937 (11,114)	1862 (2598)	2075* (11,274)	236	3932 (6944)	2566 (5357)	1366** (8810)
731–1095 days after new CV event	350	1611 (3781)	1089 (2098)	522** (4311)	210	2193 (4942)	1479 (4722)	714*** (6912)
Event type: unstable angina pectoris								
365–1 days before new CV event	581	2434 (4185)	1427 (2578)	1007* (4186)	226	1077 (989)	1123 (1351)	-46 (1608)
0–365 days after new CV event	581	9363 (7368)	941 (2575)	8422* (7761)	226	9080 (6399)	1107 (1213)	7973* (6460)
366–730 days after new CV event	523	2360 (5736)	857 (1166)	1503* (5807)	208	2103 (3850)	1212 (1301)	890** (3860)
366–1095 days after new CV event	523	4309 (7302)	1686 (2219)	2623* (7693)	208	4012 (6381)	2658 (4245)	1354** (7201)
731–1095 days after new CV event	485	2102 (4096)	894 (1559)	1208* (4447)	200	1986 (4530)	1504 (3550)	482*** (5662)
Event type: revascularization								
365–1 days before new CV event	587	2081 (3060)	1575 (4008)	506* (4593)	345	1289 (1533)	929 (951)	361 (1489)
0–365 days after new CV event	587	9506 (4316)	1002 (1545)	8504* (4530)	345	9770 (5482)	975 (848)	8795 (5581)
366–730 days after new CV event	557	1941 (3359)	868 (1165)	1073* (3577)	326	1891 (2559)	1167 (1723)	724 (3050)
366–1095 days after new CV event	557	3719 (5149)	1843 (2619)	1875* (5660)	326	3736 (4678)	2322 (3081)	1414 (5529)
731–1095 days after new CV event	528	1875 (3431)	1029 (1929)	846* (3851)	316	1904 (3173)	1192 (1762)	712 (3599)

* p value <0.01, ** p value <0.05, *** p value <0.10

Similar cost patterns for different event types were seen for the history of major CVD and CHD equivalent cohorts (Table 4). With regard to short-term costs (the 1st year after index), the order of the three most costly event types was identical in the history of major CVD and CHD risk equivalent cohorts: ischemic stroke (incremental costs for the history of major CVD and CHD risk equivalent cohort were €10,194 and €9823, respectively), myocardial infarction (€8801 and €9807), and revascularization (€8504 and €8795). Transient ischemic attack was the least costly event type for both cohorts (€3917 and €4140). A dissimilarity between the two cohorts was observed for long-term costs (731–1095 days after index) as heart failure was associated with almost twice as high costs in the history of major CVD cohort as in the CHD risk equivalent cohort (€2344 vs €1220). Heart failure was, however, the most costly event type for both cohorts in the long term.

### Subsequent events—health resource utilization and associated costs

Subsequent events were common amongst the study population, with 33 % of patients experiencing a second event within 2 years after index. Patients who had heart failure as an index event had by far the highest frequency of experiencing a subsequent event (43 %), with heart failure being the most common event type (33 % of the patients who experienced heart failure as an index event had a subsequent heart failure hospitalization). This is in line with the finding that heart failure was associated with the highest long-term costs. Conversely, revascularizations were associated with the lowest frequency of a subsequent event, with 77 % of patients with revascularization as an index event experiencing no subsequent event during 2 years following the procedure. This also aligns with revascularizations being associated with comparatively lower costs in the long term.

The healthcare costs of patients who experienced at least two new CV events were evaluated for patients in all cohorts (*n* = 4514). The index date in these analyses was set to the date of the second CV event. The pre-index costs were thus high (€6396) since they included some costs from the first CV event. Despite the high pre-index costs, a sharp increase was still observed after the second event (acute costs during the 1st month after the second event were €6416). Costs gradually decreased during subsequent years after the second event (€3271 and €2768 for the 2nd and 3rd year, respectively) but remained above levels observed for cases after the first new CV event (Table 3).

Corresponding analyses for patients who experienced at least three new events during time of exposure were also performed (*n* = 1741). The index date was set to the date of the third event in these analyses. The pre-index costs were even higher among these patients (€9879), but the short-term costs after the third new CV event were still similar to those observed after the first or second new event (acute costs during the 1st month after the second event was €6457). In the longer-term, costs decreased quite rapidly but remained at higher levels compared to patients with one or two new CV events (€3715 and €3224 for the 2nd and 3rd year, respectively).

## Discussion

There are several studies that have examined costs of CV events with a focus on short-term costs and/or first CV event. However, data are limited for long-term costs and especially for patients experiencing a subsequent CV event. There are also limited data that have focused on cost of CVD-related events over the acute phase, short term and long term for hyperlipidemia patients specifically.

This study demonstrated the substantial acute and short-term costs associated with new CV events, thereby confirming results by recent studies on the economic burden of CV events [3, 4, 6, 10]. The increase in costs just after the index event was observed regardless of patients’ CV risk level at study inclusion. Confounders are minimized by applying a matched control design and focusing on incremental costs between patients with new CV events and patients without new events. It should be borne in mind when interpreting the results that the observed HRU and associated cost of the controls are largely driven by study design and cohort definitions. For instance, patients in the history of major CVD cohort were required to have an event within 5 years of study inclusion, after which the patients defined as controls did not experience another event during the exposure time. These definitions have likely affected the observed cost pattern for controls in the history of major CVD cohort.

The results also indicated that a patient’s first CV event leads to substantial long-term costs, as only the history of major CVD cohort returned to levels close to those seen in the pre-index period 2–3 years after the index date, with costs remaining elevated in the CHD risk-equivalent and low/unknown risk cohorts. The finding that greater CV risk-level at baseline resulted in higher long-term costs is consistent with recent studies which have shown that follow-up costs after a CV event are greater if patients had a comorbidity such as diabetes before the new event [3, 11].

The study also presents healthcare costs associated with CVD-related events stratified by specific event types, which only a few studies have examined before. The findings of the study demonstrate the variation in healthcare cost of different types of events, both in the short term and in the long term. An older study by Zethraeus et al. [13] on CHD and stroke also found a variation in costs by event type and that ischemic stroke was the event type associated with the highest short-term costs which is in line with the results from this study. Transient ischemic attack was associated with the lowest short-term costs in this study, across all risk cohorts.

Of all studied event types, heart failure seemed to be associated with the greatest risk of experiencing a subsequent event and the highest long-term costs, while revascularization procedures were associated with the lowest risk of experiencing a subsequent event and lowest long-term costs. One third of the patients with new CV events had at least one subsequent event during a 2-year period after the index event.

The high representativeness, large samples, long follow-up, and data quality that underlie the analyses of HRU and costs are important strengths of this study. Sweden is well known for having excellent registers with a high degree of validity and completeness that allow for generating real-world evidence of high quality. The combination of breaking down HRU into categories and into costs over the acute phase, short term, and long term, together with the stratification of patients by risk are further strengths, as this contributes to filling important evidence gaps in the literature.

Apart from the advantages mentioned, this study has some limitations. One of them is the lack of a natural index date for controls. This meant that cases and controls were matched based on their characteristics at study inclusion, not at start of follow-up (i.e. CV event index date) when the comparison of cases and controls actually starts. This discrepancy may have led to matched pairs being less comparable at index date than they were at the time of matching. There was some evidence of this as cases became more comorbid during the time from study inclusion to index than did the controls. Thus, despite the stratification and matching of patients aimed at minimizing bias, there may have been potential confounding variables that were not controlled for in the study.

An additional limitation of the study may arise from the study population selection, as hyperlipidemia patients were included in the study based on filled prescriptions for LLT, rather than diagnoses. This was considered to be the most accurate way of identifying hyperlipidemia patients in Sweden as a diagnosis of hyperlipidemia could have been made prior to the date of first available data. However, patients with LLT prescriptions but without hyperlipidemia may still have been included. Additionally, patients without LLT but prior history of CV events were included in the study in order to capture most patients at high risk of CV events.

Another limitation of the study is that data on healthcare provided by nursing homes and similar by the municipality was not a part of this study. Further, as HRU relating to a CV event was conservatively defined as outpatient care and hospitalizations with an accompanying primary diagnosis of a CV event, it is unlikely that all HRU resulting from a CV event (e.g., rehabilitation, all follow-up visits) were captured in the study. Thus, true healthcare utilization is likely to be underestimated in most cases. Productivity losses due to CV events were also not accounted for in the study. As a previous Swedish study has found that indirect costs are of the same magnitude as the direct costs needed for treatment and rehabilitation [13], only a part of the full societal costs of CV events are captured in this study.

In conclusion, the aim of this retrospective register study was to estimate HRU and associated costs of new CV events in patients with hyperlipidemia or prior events, in the short and longer term. The study demonstrated the high costs of CV events and follow-up care up to 3 years after the CV event and contributes towards filling important gaps in available evidence by providing data on short-term (1st year) and long-term (up to 3 years) resource use and costs of new CV events for patients with varying CV risk levels, including patients with a history of CVD. The study also presents costs by specific CV event types, which provides insight into the varying economic consequences of different events. These results may serve as an important basis for further health economic analysis to predict the cost-effectiveness of new health technologies that may prevent CVD-related events.

## Electronic supplementary material

Below is the link to the electronic supplementary material.

Supplementary material 1 (PDF 100 kb)
