# Supplementary material for: Healthcare costs associated with cardiovascular events in patients with hyperlipidemia or prior cardiovascular events: estimates from Swedish population-based register data
Source: Eur J Health Econ. 2015 Jun 16;17:591–601. doi: 10.1007/s10198-015-0702-0 (PMC4869759; doi:10.1007/s10198-015-0702-0)
Supplement: Supplementary file 1 — Supplementary material 1 (PDF 100 kb) [file 10198_2015_702_MOESM1_ESM.pdf]

Healthcare costs associated with cardiovascular events in patients with hyperlipidemia or prior cardiovascular events - estimates from Swedish population-based register data

- Supplementary material

Appendix A. Codes for diagnoses and medication

Table 1. Diagnoses for cohort stratification

Disease/Procedure	ICD-10 code/KVÅ code
Myocardial Infarction	I21; I22; I23
Unstable angina pectoris	I20.0
Ischemic stroke	I63; I65; I66; I67.2; I167.8
Diabetes	E10; E11; E12; E13; E14
Peripheral artery disease	I70; I71; I74
Abdominal aortic aneurysm	I71.3; I71.4
Transient ischemic attack	G45.9
Angina pectoris	I20.1-9; I25.1
Revascularization procedures	FND10-20; FNG02; FNG05; PCQ10; PCQ20; PCQ30; PCQ40; PCQ99

Table 2. Diagnoses for CV events

Disease/Procedure	ICD-10 code/KVÅ code
Myocardial Infarction	I21; I22; I23
Unstable angina pectoris	I20.0
Ischemic stroke	I63; I65; I66; I67.2; I167.8
Heart failure	I50
Transient ischemic attack	G45.9
Revascularization procedures	FND10-20; FNG02; FNG05; PCQ10; PCQ20; PCQ30; PCQ40; PCQ99

Table 3. Charlson Comorbidity Index

Disease	ICD-10 code	Score
Myocardial Infarction	I21; I22; I23	1
Congestive Heart Failure	I50; I11.0; I13.0; I13.2	1
Peripheral Vascular Disease	I70; I71; I72; I73; I74; I77	1
Cerebrovascular Disease	I60-I69; G45; G46	1
Dementia	F00-F03; F05.1; G30	1
Chronic Pulmonary Disease	J40-J47; J60-J67; J68.4; J70.1; J70.3; J84.1; J92.0; J96.1; J98.2; J98.3	1
Connective Tissue Disease	M05; M06; M08; M09; M30; M31; M32; M33; M34; M35; M36; D86	1
Ulcer Disease	K22.1; K25-K28	1
Mild Liver Disease	B18; K70.0; K70.3; K70.9; K71; K73; K74; K76.0	1
Diabetes Mellitus		1
Insulin dependent	E10.0; E10.1; E10.9	
Non-Insulin dependent	E11.0; E11.1; E11.9	

Hemiplegia or paraplegia	G81; G82	2
Moderate-Severe Renal Disease	I12; I13; N00-N05; N07; N11; N14; N17-N19; Q61	2
Diabetes Mellitus with End Organ Damage<br>Insulin dependent<br>Non-Insulin dependent	 <br>E10.2-E10.8<br>E11.2-E11.8	2
Any Tumor	C00-C75	2
Leukemia	C91-C95	2
Lymphoma	C81-C85; C88; C90; C96	2
Moderate-Severe Liver Disease	B15.0; B16.0; B16.2; B19.0; K70.4; K72; K76.6; I85	3
Metastatic Solid Tumor	C76-C80	6
AIDS	B21-B24	6
